# XGB-AGMoE: A Validation-Adaptive XGBoost-Anchored Granular Mixture-of-Experts Framework for Multi-Class Intrusion Detection in IoMT WiFi–MQTT Traffic

**DOI:** 10.3390/s26196096

**Published:** 2026-09-25

**Authors:** Madallah Alruwaili, Fawaz J. Alruwaili, Mahmood Mohamed

**Affiliations:** 1 Department of Computer Engineering and Networks, College of Computer and Information Sciences, Jouf University, Sakaka 72388, Aljouf, Saudi Arabia; 2 Department of Computer Science, College of Computer and Information Sciences, Jouf University, Sakaka 72388, Aljouf, Saudi Arabia; fawazjr@ju.edu.sa; 3 Information Systems & Technology Department, Faculty of Graduate Studies for Statistical Research, Cairo University, Giza 12613, Egypt; mahmoodissr@cu.edu.eg

**Keywords:** granular computing, mixture-of-experts, probability calibration, multi-class intrusion detection, Internet of Medical Things (IoMT), WiFi–MQTT network traffic

## Abstract

The Internet of Medical Things (IoMT) environment is based on WiFi and MQTT communication, which results in highly imbalanced intrusion-detection data with a high degree of heterogeneity. In this study, XGB-AGMoE is proposed, a validation-adaptive XGBoost-anchored Granular Mixture-of-Experts framework that combines the quantile-derived granular descriptors and experts of CatBoost, LightGBM, XGBoost, class-wise post hoc sigmoid calibration, leakage-free logistic-regression stacking, and an XGBoost-anchored probability fusion stage. The official test partition was set aside for final evaluation after disjoint development subsets were used for preprocessing, calibration, stacking, and anchor selection. The value of 0.80 was chosen for the anchor weight for the calibrated XGBoost, and 0.20 was chosen for the calibrated stack for the canonical seed-42 experiment. The resulting model achieved 0.993887 accuracy, 0.993681 weighted-F1, 0.851256 macro-F1, 0.999634 macro-ROC-AUC, and 0.921352 macro-PR-AUC on a 150,000-record official test sample. The Brier score, negative log-likelihood, and expected calibration error were 0.007531, 0.015309, and 0.001775, respectively. Across seeds 42, 52, and 62, accuracy was 0.9926±0.0012 and macro-F1 was 0.8238±0.0251. Recall was higher for the top denial of service classes and lower for DDoS Publish Flood and Recon VulScan. The results corroborate with expert anchoring and validation-controlled evaluation; they also highlight that there are fusion gains that depend on the data split and that they should be evaluated in the light of robust component baselines.

## 1. Introduction

The Internet of Medical Things (IoMT) is a network of medical devices that are integrated with clinical information systems through wearable sensors, bedside monitors, infusion systems, medical gateways and other networked devices. While WiFi and Message Queuing Telemetry Transport (MQTT) offer efficient means of monitoring and sharing data, they also increase the attack surface area of healthcare networks. The following insecure practices can allow for spoofing, reconnaissance, denial of service, and propagation of malware: weak authentication, compromised endpoints, misconfigured gateways, and insecure publish-subscribe communication. In addition to traditional information-technology security, intrusion detection has been extended to the IoMT context due to the potential for these events to impact access to clinically important information and connected services [1,2,3,4].

There are several challenges in modeling IoMT network telemetry. Traffic types differ between devices and communication types; the attack category is also highly imbalanced. In some attacks, it is possible to identify specific high-volume patterns, while reconnaissance and application-level attacks can be indistinguishable from legitimate traffic. Nonlinear relationships in tabular traffic data can be modeled using machine learning techniques, but high accuracy on the majority of traffic types does not necessarily mean that a minority class can be accurately detected or that reliable probability estimates can be made [5,6,7,8]. For structured intrusion-detection data, gradient-boosting techniques like XGBoost, LightGBM and CatBoost are very effective for capturing the relationships between features that are nonlinear and have flexible regularization options [9,10,11,12,13].

Where predicted scores are used to prioritize alerts or as thresholds to guide responses, probability quality is especially significant. The confidence value should be consistent with the frequency of correct predictions; otherwise, classification accuracy may be high with threshold-based decisions, but they may be misleading. There are some post hoc calibration methods that can enhance the agreement between predicted confidence and empirical correctness, but calibration needs to be estimated from data that are independent of the model training and final testing [14,15,16,17].

In this paper, we propose an XGB-AGMoE framework with XGBoost as a validation-adaptive granular mixture-of-experts method for 14-class intrusion detection in IoMT WiFi–MQTT traffic. The framework extends standardized traffic variables with quantile-specific granular traffic descriptors, trains heterogeneous CatBoost, LightGBM and XGBoost experts, calibrates their probabilities using leakage-free out-of-fold predictions, and fits a logistic meta-learner with leakage-free out-of-fold predictions. Then a convex combination is performed on top of the stacked posterior using a validation-selected convex combiner, which “anchors” the posterior to calibrated XGBoost. The study provides the following novel contributions: (1) a train-fitted granular representation, describing feature position, bin confidence and empirical frequency; (2) a leakage-controlled calibration and stacking protocol; (3) a validation-adaptive anchoring mechanism that preserves the strongest component as an admissible endpoint; and (4) an evaluation of class-wise discrimination, calibration, uncertainty, repeated seeds, bootstrap confidence intervals, and inference efficiency.

## 2. Related Work

The creation of IoT-focused intrusion-detection datasets has led to testing in a more representative environment than typical enterprise-network datasets. It is demonstrated by N-BaIoT that network behavior changes can be used to identify compromised IoT devices [1]. Ashraf et al. analyzed real-time intrusion detection with the Bot-IoT benchmark and various machine learning classifiers [2], and TON_IoT integrated network traffic and telemetry from diverse IoT and industrial-IoT sources [3]. There are, however, ongoing issues with class imbalance, aging traffic patterns, heterogeneous data preprocessing, and evaluation methods allowing overfitting on the specific dataset in the surveys of intrusion-detection datasets [4,5,6,7].

Tree-based models still serve as good baselines for tabular IDS. The main problem solved by the Random Forest and related techniques is when discriminative traffic features are available, the results being competitive [8]. XGBoost is an implementation of sequential residual correction and regularization, which has been widely used for many security problems involving multiple classes [9]. LightGBM boosts computation efficiency by using histogram-based learning and leaf-wise tree building [10], while CatBoost mitigates prediction bias by employing ordered boosting [11]. The use of combinations of complementary learners has been shown to be effective in improving intrusion detection (ID) according to ensemble studies, but the improvement is contingent upon the way they are generated and fused [12,13].

The base predictions are combined in stacked generalization, using a learned meta-model [18], and the use of specialized learners with complementary inductive biases is formalized by mixture-of-experts methods [19]. These techniques are applicable to IoMT traffic since an attack family can be spread across different areas of the feature space. For meta-learners, however, it is necessary to use out-of-fold predictions for training. If the stacker is trained from the outputs of experts from the in-sample set, it will be exposed to optimistically biased probabilities, and the final evaluation will not be as valid.

A relatively less discussed aspect of intrusion detection analysis is that of probability calibration. When adequate calibration data are available, isotonic regression maps a nonparametric transformation of the model scores, while Platt scaling learns a parametric sigmoid transformation of the model scores [14,15]. However, previous research has revealed that reliable confidence estimates do not necessarily follow from strong discrimination [16,17]. Hence, in addition to discrimination measures, one should use measures like Brier score, negative log-likelihood, expected calibration error or reliability diagrams to evaluate calibrated IDS probabilities.

Granular computing is a set of observations in terms of information granules and intervals and other interval-dependent abstractions [20,21]. Granular descriptors are a great way to augment continuous values in network telemetry to express whether an observation is from a common, rare, central or boundary region of the training distribution. The existing work did not integrate train-fitted granular descriptors, heterogeneous boosting experts, held-out multi-class calibration, leakage-free stacking and validation-controlled anchoring into a single evaluation under the umbrella of CICIoMT2024. XGB-AGMoE was created to fill this methodological gap.

## 3. Proposed XGB-AGMoE Framework

Figure 1 shows that the proposed XGBoost-Anchored Granular Mixture-of-Experts (XGB-AGMoE) framework is a validation-adaptive ensemble for 14-class IoMT WiFi-MQTT intrusion detection. Firstly, numeric traffic features are aligned and filtered, followed by fitting numeric standardization and quantile transformations with development data only and the creation of numeric continuous feature views and granular feature views. CatBoost, LightGBM, and XGBoost then learn complementary decision functions. A logistic-regression meta-learner is used to combine the posterior vectors generated by each expert, using held-out calibration data to independently calibrate each expert’s sigmoid. The last step secures the fused posterior to calibrated XGBoost with a convex combination of the posterior, selected for validation. This design enables the stack to only contribute to the prespecified validation objective and leaves pure calibrated XGBoost as an admissible endpoint.

### 3.1. Model Overview and Design Rationale

A new framework for multi-class intrusion detection in the Internet of Medical Things (IoMT) network, called XGB-AGMoE, is proposed based on the XGBoost algorithm and the granular mixture-of-experts approach. The architecture is developed to cope with three properties of the CICIoMT2024 WiFi-MQTT data, as outlined below.

XGB-AGMoE is composed of four major elements, as shown in Figure 1: (1) two types of continuous and granular feature representations; (2) three types of experts (CatBoost, LightGBM, and XGBoost); (3) probability calibration is conducted on the held-out dataset and then followed by out-of-fold stacking without leakage; and (4) validation-adaptive fusion of the stacked posterior and a calibrated XGBoost anchor.

Let the cleaned dataset be

(1) $$D={\left\{(x_{i},y_{i})\right\}}_{i=1}^{N},$$

where $x_{i}\in R^{d}$ is the feature vector of sample *i*, $y_{i}\in \left\{1,\ldots ,C\right\}$ is its class label, d=44 is the number of retained numeric features, and C=14 is the number of traffic classes. The objective is to estimate a normalized class-posterior vector

(2) $$p_{i}^{\mathrm{final}}=\left[p_{i1},p_{i2},\ldots ,p_{iC}\right],\;\sum_{c=1}^{C}p_{ic}=1,$$

and obtain the final prediction as

(3) $${\hat{y}}_{i}=\arg\max_{c}\;p_{ic}^{\mathrm{final}}.$$

The key methodological contribution is not just an average of three boosting classifiers. Instead, XGB-AGMoE learns a stacking function for each class from only out-of-fold predictions and then adapts the prediction to calibrated XGBoost probabilities based on a weight from the validation partition only.

### 3.2. Leakage-Controlled Data Partitioning

The training and test files provided are used as different data source files. The original test file is not used to preprocess the estimation, to calibrate probabilities, to fit the meta-learner, or to select the fusion weights and is stored for the final evaluation, as shown in Table 1 and Algorithm 1.

There were 7,160,831 rows in the original training parquet and 1,614,182 rows in the test parquet provided. With seed 42, 15% of the initial training parquet was initially allocated to validation. The remaining 85% of the data was then split into 10 percent for calibration and 90 percent for base training, which is about 8.5% and 76.5% of the original training data, respectively. The base-training data consisted of 500,000 records in the stratified computational caps, 80,000 records in the calibration-pool computational caps, and 100,000 records in the validation caps, with 150,000 records from the separate official test parquet. Stratified caps of 50,000 records were used for each of the calibrators from the calibration pool. This was repeated with seeds 52 and 62.
**Algorithm 1** Leakage-Controlled Training of XGB-AGMoE**Require:** 
Original training data $D_{\mathrm{train}}$, separate official test data $D_{\mathrm{test}}$, number of granular bins B=8, number of OOF folds K=3, candidate anchor weights A={0.0,0.1,…,1.0}, random seed s=42.**Ensure:** 
Frozen XGB-AGMoE model M, selected anchor weight $\alpha^{*}$.  1:Align numeric columns shared by $D_{\mathrm{train}}$ and $D_{\mathrm{test}}$.  2:Remove identifier-like and constant features.  3:Replace non-finite values and encode the 14 target classes.  4:Stratify $D_{\mathrm{train}}$ into $D_{\mathrm{base}}$, $D_{\mathrm{cal}}$, and $D_{\mathrm{val}}$.  5:Preserve $D_{\mathrm{test}}$ for final evaluation only.  6:Fit the standardization transformation on $D_{\mathrm{base}}$.  7:Fit the quantile granularizer on standardized $D_{\mathrm{base}}$.  8:Construct continuous view $X^{s}$, granular view G, and mixed view $\left[X^{s}|G\right]$.  9:Compute balanced sample weights from the labels in $D_{\mathrm{base}}$.10:**for** each expert e∈{CatBoost,LightGBM,XGBoost} **do**11:   Train *e* on $D_{\mathrm{base}}$ using its assigned feature view.12:   Fit an OVR sigmoid calibrator using $D_{\mathrm{cal}}$.13:   Produce calibrated probabilities for $D_{\mathrm{val}}$.14:**end for**15:Initialize empty out-of-fold probability matrices.16:**for** each stratified OOF fold k=1,…,K **do**17:   Divide the fold-development data into fold-fit and fold-calibration subsets.18:   Refit the scaler and granularizer using fold-fit only.19:   Train the three experts on fold-fit.20:   Fit fold-specific calibrators on fold-calibration data.21:   Predict the untouched fold holdout.22:   Store calibrated OOF probabilities.23:**end for**24:Construct the OOF meta-features from expert probabilities, maximum confidence, predictive entropy, and expert disagreement.25:Fit a class-balanced multinomial logistic stacker using the OOF meta-features.26:Apply the fitted stacker to the calibrated validation probabilities to obtain $p_{\mathrm{stack},\mathrm{val}}$.27:**for** every α∈A **do**28:   Compute $p_{\mathrm{final},\mathrm{val}}\left(\alpha \right)=\mathrm{Normalize}\left[\alpha p_{\mathrm{XGB},\mathrm{val}}+\left(1-\alpha \right)p_{\mathrm{stack},\mathrm{val}}\right]$.29:   Calculate validation macro-F1.30:**end for**31:Select the value $\alpha^{*}$ with the highest validation macro-F1.32:Freeze the schema, scaler, granularizer, experts, calibrators, stacker, and $\alpha^{*}$.33:**return** The frozen model M and $\alpha^{*}$.

The preprocessing audit selected 44 numeric features, dropped one constant feature, and found no identifier-like columns under the specified automatic rules. Development data only were used for standardization and quantile transformations. Auditing for preprocessing yielded 44 numeric predictors and removed one feature with an unacceptably low training variance (of less than $10^{-12}$). Prior to model fitting, column names were normalized and compared to explicit rules for IP addresses, MAC addresses, timestamps, dates, flow ID, session ID, connection ID, packet ID, UUID, and other related variants. No common numeric columns were found with these identifier rules, and no predictor was added or dropped because of the audit. Infinite values were recoded as missing and assigned a value of 0. Standardization and quantile transformations were only fitted to the base training partition and then applied identically to the calibration, validation and official test partitions.

### 3.3. Preprocessing and Continuous Feature Construction

The numeric variables that are present in both the training file and the official test file will be kept. Column names are normalized and validated for known identifiers, such as IP address, MAC address, flow number, session number, packet number, and time. These variables are excluded from the list when they are detected, as they can represent information specific to the acquisition or the connection and not a generic traffic pattern.

The values of *∞* are converted to missing and mapped to zero. All features that have variance within the training set of <10^−12^ are treated as constant and discarded. After the constant-feature removal process, 44 numeric predictors were kept from the data for the present data audit.

Each retained feature is standardized using statistics estimated exclusively from the base-training partition. For feature *j*, the standardized value is

(4) $$x_{ij}^{s}=\frac{x_{ij}-\mu_{j}^{\mathrm{base}}}{\sigma_{j}^{\mathrm{base}}+\epsilon },$$

where $\mu_{j}^{\mathrm{base}}$ and $\sigma_{j}^{\mathrm{base}}$ are the mean and standard deviation calculated from the base-training observations. The resulting continuous representation is denoted by

(5) $$X^{s}\in R^{N\times d}.$$

The same frozen transformation is subsequently applied to the calibration, validation, and official test partitions.

### 3.4. Granular Feature Construction

A granular representation is constructed to describe not only the standardized value of each feature but also its position and local support within the empirical training distribution. This representation is fitted only on the base-training partition.

For each standardized feature $x_{ij}^{s}$, a quantile transformation maps the feature to an approximately uniform variable:

(6) $$u_{ij}=Q_{j}\left(x_{ij}^{s}\right),\;u_{ij}\in \left[0,1\right],$$

where $Q_{j}\left(\cdot \right)$ is learned from the base-training data. The implementation uses at most 512 empirical quantiles and a fitting subsample of at most 200,000 observations.

The transformed interval is divided into B=8 equal-width granular bins. The bin occupied by $u_{ij}$ is

(7) $$b_{ij}=\mathrm{clip}\left(\left\lfloor Bu_{ij}\right\rfloor ,0,B-1\right).$$

Three descriptors are then computed for every original feature.

#### 3.4.1. Normalized Bin Identity

The normalized bin identifier represents the relative position of an observation within the empirical feature distribution:

(8) $$g_{ij}^{\mathrm{id}}=\frac{b_{ij}}{B-1}.$$

Values close to zero indicate placement in the lower part of the distribution, whereas values close to one indicate placement in its upper part.

#### 3.4.2. Granular Confidence

Let the center of bin $b_{ij}$ be

(9) $$m_{ij}=\frac{b_{ij}+0.5}{B}.$$

The granular confidence is defined by the normalized distance between $u_{ij}$ and its bin center:

(10) $$g_{ij}^{\mathrm{conf}}=1-\min\left(\frac{\left|u_{ij}-m_{ij}\right|}{0.5/B+\epsilon },1\right).$$

A value near one means that the observation lies close to the center of its assigned granule. A value near zero indicates proximity to a bin boundary and, therefore, greater ambiguity in its granular assignment.

#### 3.4.3. Empirical Bin Frequency

The frequency descriptor measures the training support of the assigned granule:

(11) $$g_{ij}^{\mathrm{freq}}=\frac{\sum_{n\in D_{\mathrm{base}}}I\left(b_{nj}=b_{ij}\right)}{\left|D_{\mathrm{base}}\right|}.$$

A low frequency identifies a relatively unusual region of the feature distribution, while a high frequency indicates a commonly observed traffic pattern.

The complete granular representation is

(12) $$G_{i}=\left[g_{i}^{\mathrm{id}}\;∥\;g_{i}^{\mathrm{conf}}\;∥\;g_{i}^{\mathrm{freq}}\right]\in R^{3d},$$

where || denotes vector concatenation. Because d=44, the granular representation contains 132 variables. The mixed representation supplied to CatBoost is

(13) $$X_{i}^{\mathrm{mix}}=\left[X_{i}^{s}\;∥\;G_{i}\right]\in R^{4d},$$

which produces 176 input variables.

The interpretation of this design is that $X^{s}$ preserves precise continuous measurements, whereas G supplies distribution-aware context. This allows the model to distinguish between numerically similar values located in common and uncommon regions of the training distribution.

### 3.5. Heterogeneous Boosting Experts

XGB-AGMoE uses three heterogeneous gradient-boosting experts:

(14) $$E=\left\{E_{\mathrm{Cat}},E_{\mathrm{LGB}},E_{\mathrm{XGB}}\right\}.$$

The experts use different tree-construction and regularization mechanisms, which encourage diversity in their class-probability estimates.

#### 3.5.1. CatBoost Expert

CatBoost receives the mixed continuous–granular representation:

(15) $$q_{i}^{\mathrm{Cat}}=E_{\mathrm{Cat}}\left(X_{i}^{\mathrm{mix}}\right).$$

It is configured with 300 boosting iterations, depth 6, learning rate 0.09, and $L_{2}$ leaf regularization of 5. CatBoost is assigned the mixed view because its ordered boosting procedure can exploit interactions between the original measurements and the constructed granular descriptors.

#### 3.5.2. LightGBM Expert

LightGBM operates on the standardized continuous representation:

(16) $$q_{i}^{\mathrm{LGB}}=E_{\mathrm{LGB}}\left(X_{i}^{s}\right).$$

The expert uses 500 trees, 63 leaves, a learning rate of 0.06, row and column subsampling rates of 0.85, $L_{2}$ regularization of 2.0, $L_{1}$ regularization of 0.4, and a minimum of 150 observations per leaf.

Its leaf-wise growth strategy can efficiently capture localized decision regions, while the minimum-leaf and regularization constraints reduce overfitting to rare traffic configurations.

#### 3.5.3. XGBoost Expert and Performance Anchor

XGBoost also operates on the standardized continuous representation:

(17) $$q_{i}^{\mathrm{XGB}}=E_{\mathrm{XGB}}\left(X_{i}^{s}\right).$$

It uses 500 histogram-based trees, depth 6, learning rate 0.06, maximum bin count 128, row and column subsampling rates of 0.85, $L_{2}$ regularization of 1.5, and $L_{1}$ regularization of 0.3.

XGBoost is additionally used as the performance anchor in the final fusion stage. This does not remove the other experts. Instead, it allows the final decision to retain the strongest stable probability component while incorporating complementary class-dependent information learned by the mixture-of-experts stack.

To reduce bias toward majority classes, balanced sample weights are used when fitting all three base experts:

(18) $$w_{c}=\frac{N}{C\;N_{c}},$$

where $N_{c}$ is the number of base-training observations belonging to class *c*.

### 3.6. Held-Out Per-Expert Probability Calibration

The raw probability vectors produced by boosted trees may be accurate for classification but can still be overconfident or underconfident. XGB-AGMoE therefore calibrates each expert independently using the disjoint calibration partition.

For expert *e* and class *c*, the raw probability $q_{iec}$ is first clipped and converted to a logit:

(19) $$\ell_{iec}=\log\left(\frac{{\tilde{q}}_{iec}}{1-{\tilde{q}}_{iec}}\right),$$

where

(20) $${\tilde{q}}_{iec}=\mathrm{clip}\left(q_{iec},\epsilon ,1-\epsilon \right).$$

A one-versus-rest sigmoid mapping is fitted:

(21) $$s_{iec}=\sigma \left(a_{ec}\ell_{iec}+b_{ec}\right),$$

where $a_{ec}$ and $b_{ec}$ are learned using binary logistic regression and σ(·) is the sigmoid function. The independently calibrated class scores are then renormalized:

(22) $$p_{iec}=\frac{s_{iec}}{\sum_{r=1}^{C}s_{ier}}.$$

Calibration uses a stratified subset of at most 50,000 observations. Class weighting is not applied to this stage so that the calibrated probabilities preserve the empirical class prior rather than an artificially balanced prior.

Because of its low-variance parametric mapping and relative stability of the mapping when the number of calibration observations in some attack classes is small, one-versus-rest sigmoid calibration was chosen. A nonparametric approach that allows more flexibility, like isotonic regression, can overfit calibration subsets that are small for the minority class and yield irregular mappings. Each expert’s confidence scale is also corrected independently for each class. The resulting probability vectors after those transformations are not necessarily in a multi-class probability simplex, so they are renormalized to ensure all class probabilities are non-negative and sum to 1.

### 3.7. Leakage-Free Out-of-Fold Stacking

Directly fitting a meta-classifier on predictions generated from the same observations used to train the experts would produce optimistic and potentially unstable results. Therefore, XGB-AGMoE constructs its meta-training data using three-fold stratified out-of-fold prediction.

The base-training partition is divided into K=3 folds. For fold *k*, the experts are trained using the fold-development observations. A further disjoint subset containing 10% of the fold-development observations is reserved for fold-specific calibration. The fitted experts and their calibrators are then applied to the fold holdout.

Consequently, every base-training observation receives predictions from models that did not use that observation for training or calibration.

For each sample, the calibrated probabilities of the three experts are concatenated:

(23) $$z_{i}^{p}=\left[p_{i}^{\mathrm{Cat}}\;∥\;p_{i}^{\mathrm{LGB}}\;∥\;p_{i}^{\mathrm{XGB}}\right].$$

The meta-representation also incorporates each expert’s maximum confidence:

(24) $$m_{ie}=\max_{c}\;p_{iec},$$

its predictive entropy:

(25) $$h_{ie}=-\sum_{c=1}^{C}p_{iec}\log\left(p_{iec}+\epsilon \right),$$

and the disagreement among the three expert confidence values:

(26) $$d_{i}=\mathrm{Std}\left(m_{i,\mathrm{Cat}},m_{i,\mathrm{LGB}},m_{i,\mathrm{XGB}}\right).$$

The final meta-feature vector is

(27) $$z_{i}=\left[z_{i}^{p}\;∥\;m_{i,\mathrm{Cat}},m_{i,\mathrm{LGB}},m_{i,\mathrm{XGB}}\;∥\;h_{i,\mathrm{Cat}},h_{i,\mathrm{LGB}},h_{i,\mathrm{XGB}}\;∥\;d_{i}\right].$$

For 14 classes, this produces 3C+7=49 meta-features. A class-balanced multinomial logistic-regression model with $C_{\mathrm{reg}}=1.0$ and a maximum of 1000 iterations is fitted to the out-of-fold meta-features:

(28) $$p_{i}^{\mathrm{stack}}=\mathrm{softmax}\left(Wz_{i}+b\right).$$

This meta-learner does more than assign one global weight to each expert. It learns class-specific relationships between expert probabilities, confidence, entropy, and disagreement. It can therefore emphasize different experts for different attack classes or uncertainty conditions.

### 3.8. Validation-Adaptive XGBoost-Anchored Fusion

Although stacking captures complementary expert behavior, it can be affected by noisy minority-class probabilities or distribution differences between the out-of-fold and final fitted models. XGB-AGMoE consequently introduces an adaptive residual connection from the calibrated XGBoost expert to the stacked posterior.

For each validation sample, candidate final probabilities are computed as

(29) $$p_{i}^{\mathrm{final}}\left(\alpha \right)=\mathrm{Normalize}\left[\alpha p_{i}^{\mathrm{XGB}}+\left(1-\alpha \right)p_{i}^{\mathrm{stack}}\right],$$

where

(30) α∈A={0.0,0.1,0.2,…,1.0}.

The optimal anchor weight is selected exclusively on the validation partition:

(31) $$\alpha^{*}=\arg\max_{\alpha \in A}MacroF1\left(y^{\mathrm{val}},{\hat{y}}^{\mathrm{val}}\left(\alpha \right)\right).$$

The validation experiment selected

(32) $$\alpha^{*}=0.80,$$

which assigns 80% of the final probability mass to the calibrated XGBoost anchor and 20% to the calibrated stacked posterior:

(33) $$p_{i}^{\mathrm{final}}=\mathrm{Normalize}\left[0.80p_{i}^{\mathrm{XGB}}+0.20p_{i}^{\mathrm{stack}}\right].$$

The selected value indicates that XGBoost supplies the principal discrimination component, while the stacked mixture provides a controlled corrective contribution. The stacked component is therefore retained only to the extent supported by validation performance. This validation-adaptive mechanism prevents the model from assuming that an equal-weight ensemble or an unconstrained stack must always be superior.

After selecting $\alpha^{*}$, the weight is frozen. No fusion parameter is modified using the official test labels.

Suppose that $p_{\mathrm{XGB}}\left(x\right)$ is the vector of probabilities that the XGBoost model expects from the sample *x* and $p_{\mathrm{stack}}\left(x\right)$ is the vector of probabilities that the logistic-stacked model expects from sample *x*. The final posterior is given by

(34) $$p_{\mathrm{final}}\left(x;\alpha \right)=\mathrm{Normalize}\left[\alpha p_{\mathrm{XGB}}\left(x\right)+\left(1-\alpha \right)p_{\mathrm{stack}}\left(x\right)\right],$$

with α∈{0.0,0.1,…,1.0}. α=0 is the original calibrated stack, while α=1 is the calibrated XGBoost model. The validation partition is used only for each candidate weight. The chosen value is the one that maximizes validation macro-F1, with validation NLL being the tie-breaker, and preference for the bigger XGBoost weight being the third criterion. The official test partition is NOT used when selecting weights.

The validation procedure chosen was α=0.80, which results in an 80% calibrated-XGBoost and 20% calibrated-stack posterior for seed 42. The selected validation macro-F1 was 0.940453, compared with 0.939951 at α=1.00. The final weight selected was then frozen and used once with the official test sample. While this small validation advantage was not enough to make the model superior to the calibrated XGBoost on the canonical test split, the adaptive fusion should hence not be considered as the best model but rather as a model that was validated.

The reported classifier configurations were used for the evaluation of the official test partition and were static. Considering the official test predictions or labels, no parameter was chosen. Rather than doing an exhaustive search of the hyperparameter space, the experiments used regularized, computationally practical configurations that were developed during the creation of the experiments and were then fixed for the principal experiments, repeated seeds, and ablation experiments. CatBoost employed 300 iterations, depth 6, learning rate 0.09, and regularization of 5 ($L_{2}$ leaf). LightGBM has 500 estimators, 63 leaves, a learning rate of 0.06, row subsampling of 0.85, column subsampling of 0.85, $L_{1}$ regularization of 0.4, $L_{2}$ regularization of 2.0, and min observations per leaf of 150. For XGBoost, 500 histogram-based trees with depth 6, learning rate 0.06, row and column subsampling rates of 0.85, $L_{1}$ regularization 0.3, and $L_{2}$ regularization 1.5 were used. This fixed policy guarantees that any differences between the reported configurations are due to the modeled components and not to the separate test-driven tuning.

### 3.9. Prediction Outputs and Reliability Measures

For every test observation, XGB-AGMoE returns three complementary outputs as shown in Algorithm 2. The first is the predicted traffic class obtained from the maximum final posterior probability. The second is the calibrated confidence:

(35) $${\mathrm{Conf}}_{i}=\max_{c}\;p_{ic}^{\mathrm{final}}.$$

The third is predictive entropy:

(36) $${\mathrm{Unc}}_{i}=-\sum_{c=1}^{C}p_{ic}^{\mathrm{final}}\log\left(p_{ic}^{\mathrm{final}}+\epsilon \right).$$

High confidence and low entropy is a focused class vote. On the other hand, if the confidence level is low or the entropy is high, it means that the prediction is uncertain because the mass is spread out and should be interpreted cautiously.

The official test evaluation reports both discrimination and probability-quality reports. These are used to measure classification effectiveness—accuracy, macro-F1, and weighted-F1; class-wise ranking performance—macro-ROC-AUC and macro-PR-AUC; and reliability of final probability estimates—Brier score, negative log-likelihood, expected calibration error, and maximum calibration error.

The performance of the canonical seed-42 XGB-AGMoE model on the official test partition that was not used to validate the results above was as follows: accuracy, 0.993887; macro-F1, 0.851256; weighted-F1, 0.993681; macro-ROC-AUC, 0.999634; macro-PR-AUC, 0.921352; Brier score, 0.007531; negative log-likelihood, 0.015309; and expected calibration error, 0.001775.
**Algorithm 2** XGB-AGMoE Prediction**Require:** 
Unseen observation *x*, frozen XGB-AGMoE model M, frozen anchor weight $\alpha^{*}$.**Ensure:** 
Predicted class $\hat{y}$, calibrated confidence, predictive uncertainty.  1:Align *x* with the retained training feature schema.  2:Replace non-finite values using the fixed data rule.  3:Apply the frozen standardization transformation: $x^{s}=\mathrm{Standardize}\left(x\right)$.  4:Apply the frozen quantile granularizer: $g=\mathrm{Granularize}(x^{s})$.  5:Construct the mixed vector: $x^{\mathrm{mix}}=\left[x^{s}|g\right]$.  6:Obtain raw probabilities: $q^{\mathrm{Cat}}=\mathrm{CatBoost}\left(x^{\mathrm{mix}}\right)$, $q^{\mathrm{LGB}}=\mathrm{LightGBM}\left(x^{s}\right)$, and $q^{\mathrm{XGB}}=\mathrm{XGBoost}\left(x^{s}\right)$.  7:Apply the corresponding held-out calibrator: $p^{\mathrm{Cat}}={\mathrm{Calibrate}}_{\mathrm{Cat}}\left(q^{\mathrm{Cat}}\right)$, $p^{\mathrm{LGB}}={\mathrm{Calibrate}}_{\mathrm{LGB}}\left(q^{\mathrm{LGB}}\right)$, and $p^{\mathrm{XGB}}={\mathrm{Calibrate}}_{\mathrm{XGB}}\left(q^{\mathrm{XGB}}\right)$.  8:Construct the meta-feature vector from calibrated probabilities, maximum confidence, entropy, and disagreement.  9:Obtain the stacked posterior: $p^{\mathrm{stack}}=\mathrm{LogisticStacker}\left(\mathrm{meta}\text{-}\mathrm{features}\right)$.10:Compute $p^{\mathrm{final}}=\mathrm{Normalize}\left[\alpha^{*}p^{\mathrm{XGB}}+\left(1-\alpha^{*}\right)p^{\mathrm{stack}}\right]$.11:Predict $\hat{y}=\arg\max_{c}p_{c}^{\mathrm{final}}$.12:Compute confidence: $\mathrm{confidence}=\max_{c}p_{c}^{\mathrm{final}}$.13:Compute uncertainty: $\mathrm{uncertainty}=-\sum_{c}p_{c}^{\mathrm{final}}\log\left(p_{c}^{\mathrm{final}}+\epsilon \right)$.14:**return** $\hat{y}$, confidence, and uncertainty.

## 4. Results and Discussion

### 4.1. Dataset and Experimental Partitions

The experiments employed the WiFi–MQTT component of CICIoMT24, a multiprotocol IoMT cybersecurity dataset that was created in both real and simulated medical-device environments [22]. The evaluated taxonomy included 14 classes, of which 12 were benign traffic, spoofing, reconnaissance, malformed data, publish-flood attacks, and protocol-specific denial of service.

The provided training and test parquet files were considered as different sources. The study was not designed to split the data into an 80% train/test split. Rather, 15% of the training data provided were assigned for validation. About 76.5% of the original training data was used for base-model fitting after 10% of the remaining development data was allocated for calibration. The official test sample was only taken from the provided test parquet and not used for any preprocessing, calibration, stacking, or anchor selection process.

The official test sample was very unbalanced. The number of observations for each class varied from 46,428 (DoS UDP) to 17 (Ping Sweep), with 96 observations for Recon VulScan. Together with macro-F1, macro-PR-AUC, class-specific precision and recall, probability-calibration metrics, and uncertainty intervals, the metrics are interpreted accordingly.

### 4.2. Experimental Results

#### 4.2.1. Overall Predictive and Calibration Performance

Table 2 shows the performance of XGB-AGMoE for prediction and calibration on the official test set without any modifications. The model was able to obtain an accuracy of 0.993887 and a weighted-F1 of 0.993681, showing very accurate classification performance on the entire test distribution. The lower macro-F1 of 0.851256 suggests there was less consistency in performance across the 14 classes. The differences between the weighted and macro-averaged measures are in line with the fact that the class distribution in CICIoMT2024 is quite imbalanced and rare attack classes have minimal impact on both overall accuracy and the weighted-F1 measure while being equally weighted in the macro-F1 measure.

The macro-ROC-AUC is very close to a perfect value (0.999634), indicating that the model performs very well when ranking classes. The macro-PR-AUC of 0.921352 also shows good precision–recall performance, yet its lower score compared to ROC-AUC is due to the challenge of detecting minority classes when there is a skewed class distribution. As a result, the overall accuracy is generally higher than macro-F1 and macro-PR-AUC.

The Brier score of 0.007531 and the NLL of 0.015309 demonstrate that the posterior probabilities were mostly precise and well localized around the right classes. The average predicted confidence is very close to the empirical correctness, with an ECE of 0.001775, which is very close to the value of zero. However, the result of the MCE (0.220490) shows that one or more bins had a relatively large confidence–accuracy deviation. MCE is the largest discrepancy in the worst bin and is more likely to be affected by a sparsely populated bin, so it should be considered in conjunction with the much more modest ECE and not as proof of poor overall calibration. Together, these findings show that XGB-AGMoE provides good discrimination and good average confidence estimates, and that calibration at the worst bin and performance for minority classes could be improved.

#### 4.2.2. Class-Wise Performance and Confusion Analysis

Table 3 shows the results of XGB-AGMoE on the official test partition of 150,000 samples, presented by class. The results of the model for the well-represented denial-of-service classes were close to perfect. DoS Connect Flood, DoS ICMP, DoS SYN, DoS TCP, and DoS UDP achieved F1-scores between 0.99930 and 0.99988. The results showed that the model achieved high performance for two of the most prevalent volumetric and protocol-based attacks, learning stable and very separable patterns.

The model’s precision of 0.96304, recall of 0.94678, and F1-score of 0.95484 were also very high for benign traffic across 3495 observations. The result is significant, as it shows that the majority of legitimate connections were correctly identified as legitimate rather than as malicious traffic. Port Scan also presented a high F1-score of 0.93683, with a recall of 0.97716, even though the precision of 0.89969 suggests that some observations from other classes were mislabeled as a part of this class.

For Malformed Data and OS Scan, a more moderate performance was seen. Malformed Data had a high precision score of 0.96923, which means that the actual class of Malformed Data was often predicted correctly; its recall score was 0.77301, indicating about 25% of the true Malformed Data instances were classified as something else. The F1-score of 0.79268, precision of 0.86667, and recall of 0.73034 indicate that OS Scan was also conservative.

There were notable precision–recall trade-offs for several minority classes. DDoS Publish Flood’s precision was 1.00000, with a recall of 0.51151. As a result, the observations classified as DDoS Publish Flood were correct, but almost 50% of the samples in this class were not detected. DoS Publish Flood, on the other hand, had a perfect recall of 1.00000 while precision was 0.67607. This implies that the observations of all actual DoS Publish Floods were detected, with some of the samples from other classes erroneously placed in this class. These two related classes show opposing behaviors, which indicates that there is possibly still some confusion left over from attacks in their category with similar publish-flood characteristics.

The results for ARP Spoofing were 0.80864 for recall, 0.61502 for precision, and 0.69867 for the F1-score. Therefore, the model detected most ARP Spoofing observations but also a relatively high number of false-positive predictions. Notably, however, only 17 test observations were used to calculate the F1-score of Ping Sweep, so this result should be interpreted cautiously, since a few errors can significantly impact the precision, recall, and F1-score reported.

Class Recon VulScan was the most difficult class with an F1-score of 0.54422. The precision of 0.78431 means that the majority of Recon VulScan predictions were accurate, but the recall rate of 0.41667 indicates that the model was not accurate in predicting more than half of the actual observations. This behavior may be the result of only 96 samples or could be confused for other reconnaissance categories, such as OS Scan and Port Scan.

The macro-average precision, recall, and F1-score were 0.88375, 0.84769, and 0.85126, respectively. The values are also problematic because they treat each class equally regardless of its difficulty of recognition; the values expose the remaining difficulty in recognizing the rare attack types. In comparison, the weighted-average precision, recall, and F1-score were 0.99483, 0.99389, and 0.99368, respectively. The large difference between the macro and weighted results is explained by the severe class imbalance: class support ranges from only 17 Ping Sweep observations to 46,428 DoS UDP observations. The weighted averages, therefore, closely reflect the near-perfect scores of the majority DoS classes, while the macro scores are still influenced by the weaker DoS classes (ARP Spoofing, DoS Publish Flood, Ping Sweep, and Recon VulScan).

Considering the overall performance, XGB-AGMoE has shown its high reliability in traffic category classification and some clinically relevant classes of network attacks. However, the results of the class-wise analysis should not be interpreted without regard to the overall accuracy. These low F1-scores for the minority classes and the asymmetric precision-recall curves suggest that future research should focus on representing the rare classes, handling related publish-flood attacks, and being more sensitive in detecting attacks that exploit reconnaissance.

Figure 2 shows the XGB-AGMoE classification behavior in the complementary absolute and class-balanced views. The count-based matrix is highly diagonalized, indicating a high percentage of correctly classified official test observations, most of which were on the main diagonal. The results of the DoS Connect Flood, DoS ICMP, DoS Publish Flood, DoS SYN, DoS TCP, and DoS UDP diagonal concentrations were particularly strong. Most of the test observations are found within these classes; however, as a result of the large number of cells in these classes, errors affecting rare classes may be visually masked by the absolute matrix. The row-normalized matrix resolves this issue, as it normalizes each row to a value of one, allowing for the diagonal elements to be interpreted as a class-specific recall.

XGB-AGMoE achieved 100% recall for 4186 DoS Connect Flood observations and 100% recall for 24,597 DoS TCP observations. Perfect recall was obtained for the DoS Publish Flood with 791 observations properly detected. Similarly, DoS ICMP, DoS SYN, and DoS UDP achieved recalls of 0.99923, 0.99869, and 0.99976, respectively. This normalized matrix then looks almost like a diagonal matrix. The same is true of benign traffic, which also showed a very strong diagonal pattern: out of 3495 observed samples, 3309 were correctly identified with a recall of 0.94678. The highest error routes were observed towards Port Scan, with 126 observations assigned as benign, and ARP Spoofing, with 53 observations. The Port Scan was reliably recognized with 2054 of 2102 observations correctly classified and a recall of 0.97716. Its few mistakes were assigned to OS Scan (33).

The greatest degree of confusion was between the two publish-flood categories. Only 400 of these 782 true DDoS Publish Flood observations were correctly classified, while 379 were classified as DoS Publish Flood. Thus, around 48.5% of DDoS Publish Flood observations were classified as DoS Publish Flood (a similar class to Publish Flood). This confusion accounts for the relatively low DDoS Publish Flood recall of 0.51151. It also explains why DoS Publish Flood achieved perfect recall but a lower precision of 0.67607: The model identified all true DoS Publish Flood samples but also classified a significant proportion of DDoS Publish Flood observations as DoS Publish Flood.

The normalized matrix also shows the remaining challenges with minority and reconnaissance classes. With ARP Spoofing, 131 of 162 observations were correctly classified (ARP recall = 0.80864). The classification of its main error was benign, with 21 observations, or around 13.0% of the class. There were 7 more ARP Spoofing observations to Recon VulScan.

Malformed Data had 126 correct predictions on 163 observations, which gives a recall of 0.77301. The most common anomalies were 23 (benign) and 13 (ARP Spoofing). These errors indicate there were some anomalous packets that lacked distinguishing features from legitimate traffic or spoofing that were not always able to be separated.

The OS Scan made 356 predictions, of which 260 were correct, giving it a recall of 0.73034. The largest group of misclassified observations was confused with the similar Port Scan class, involving 68 observations (or about 19.1% of the class). There were 24 other OS Scan observations that were categorized as benign. This type of error pattern is acceptable in terms of features, as OS Scan and Port Scan are both reconnaissance types of activities and could potentially have similar connection and probing behavior.

Only 17 Ping Sweep observations were available, of which 12 were correctly classified. Three were classified as Benign, one as ARP Spoofing, and one as Port Scan, yielding a recall of 0.70588. The class has very little support; hence, any error in this class causes a significant amount of change in its normalized error rate. Therefore, any interpretation of its confusion pattern should be treated with great caution.

Recon VulScan was the most challenging of all classes. Of the 96 observations, it was able to recall 40, which is a recall of 0.41667. The most common misclassification was benign, in 49 observations (around 51.0% of the class). Fewer observations were classified as OS Scan, Port Scan, Malformed Data, and ARP Spoofing. The Recon VulScan observations with a small number of attacks had these strong attack characteristics in the space of features retained and therefore were grouped into the Benign class.

Overall, this is the row-normalized matrix, which gives equal visual importance to each class, and the count matrix is shows high performance in the aggregate sense. The two panels indicate that XGB-AGMoE is very reliable in the dominant DoS categories and benign traffic but struggles with distinguishing DoS Publish Flood and DoS Publish Flood and with rare reconnaissance categories, particularly Recon VulScan, Ping Sweep, and OS Scan.

#### 4.2.3. Discrimination, Calibration, and Learning-Curve Analysis

XGB-AGMoE was assessed for its class-ranking ability based on complementary one-versus-rest ROC and PR analyses in Figure 3. The curves in the ROC panel are tightly grouped around the upper left corner and still lie significantly above the random-classification line. The model’s macro-ROC-AUC was 0.9996, indicating that it nearly correctly sorted positive cases from negative cases for the 14 traffic classes.

The ROC-AUC of the five classes shown—DoS UDP, DoS ICMP, DoS SYN, DoS TCP, and DoS Connect Flood—was near 1.000 for all five classes. The result aligns well with their almost perfect class-wise precision, recall, and F1 measure. An ROC-AUC of around 0.999 was also achieved with the Benign class, which is a very high degree of separation between legitimate traffic and the amalgamated set of attack observations.

The PR panel offers a more conservative estimate, as it is directly influenced by the false-positive predictions and class prevalence. The macro average precision of the model is 0.9214 on all 14 classes. The average precision for the DoS classes was around 1.000, and the average precision for the benign class was around 0.995. The curves are also near the upper right margin for all the recall thresholds, indicating that high sensitivity can be obtained at a relatively low cost in terms of false-positive predictions.

When recall is close to maximum, a decrease in precision can be seen. This behavior suggests and confirms that the most challenging positive observations must be classified under a looser criterion, adding extra false positives. This trade-off is anticipated at high recall and is significant in determining an operating threshold for real-world deployment of intrusion detection.

It is worth noting that the difference between macro-ROC-AUC and macro average precision is 0.9996 and 0.9214, respectively. In strongly imbalanced datasets, the number of observations that are true negatives may cause the false-positive rate to be very low, resulting in a very high ROC-AUC. PR analysis does not have true negatives and is therefore more sensitive to false-positive errors and poor recognition of rare classes.

The results of the ROC analysis are in general very good, thus corroborating the excellent global discrimination, while the results of the PR analysis are more convincing regarding the performance under class imbalance. The results on the two panels are combined, revealing almost perfect separation of the DoS and Benign classes and still excellent, though less consistent, precision–recall performance over the entire 14-class taxonomy.

The reliability of five probability-combination strategies is compared in Figure 4. The ideal classification is the diagonal reference line—points on the line indicate correct classification, while points away from the line indicate incorrect classifications. Any of the points lying below the diagonal represent overconfidence, and those lying above represent underconfidence.

The proposed XGBoost-anchored GMoE resulted in the minimum ECE value of 0.0018, reflecting the best overall match between the confidence and the correctness of the prediction. This value is similar to the more accurate test value of 0.001775, as mentioned in Table 2. The calibrated GMoE stack had the second-best ECE of 0.0038, followed by the raw expert average of 0.0044, the calibrated expert average of 0.0046, and the true raw stack of 0.0052.

The model that uses XGBoost as the anchoring method decreased ECE by around 52.6% compared to the calibrated GMoE stack. It also helped to lower ECE by ∼59.1% compared to the raw expert average, ∼60.9% compared to the calibrated expert average, and ∼65.4% compared to true raw stacking. The reductions illustrate that the reliability of the final posterior probabilities was not only enhanced in terms of discrimination but also enhanced overall.

The calibrated GMoE stack was better calibrated than true raw stacking, bringing ECE down from 0.0052 to 0.0038. This improvement supports the use of held-out per-expert sigmoid calibration prior to fitting the probability-level meta-learner. But simply taking the average of the experts did not yield benefits in terms of ECE: The calibrated expert average resulted in an ECE of 0.0046, while the raw expert average led to an ECE of 0.0044. This observation demonstrates that an unlearned equal-weight combination of individual experts is not necessarily optimally calibrated given the calibration of the individual experts.

The low- and intermediate-confidence regions exhibit significant differences in reliability curves. Some bins are considerably higher or lower than the diagonal. For instance, if points fall above the diagonal, this means that these predictions are conservative and the empirical accuracy was better than the reported confidence level; if points fall below the diagonal, this means that the local confidence was too high. These variations should be interpreted cautiously, as XGB-AGMoE is a very accurate classifier, and most of the predictions are in the highest confidence bins. The lower confidence bins thus have relatively few data points and hence are more vulnerable to errors in individual data points.

This separation also accounts for how XGB-AGMoE is able to have such a low ECE (0.001775) with such a high MCE (0.220490). ECE is a frequency-weighted average of the confidence–accuracy gap in each bin and is thus heavily influenced by the more populous high-confidence bin. MCE only looks at the largest deviation from any given individual bin, so if that bin has relatively few observations, it will not be included in MCE. The resulting higher MCE suggests that there is a large difference between the worst bin and the rest of the test distribution, not that the calibration is bad all over the distribution.

The XGBoost-anchored curve tends to the perfect-calibration line in the high-confidence region and ends near (1,1), reflecting that predictions that were infused with very high levels of confidence were accurate at a similarly high rate. The region is operationally significant, as it provides the majority of test observations and thus has the biggest impact on the overall probability quality rating.

In general, the comparison suggests that equal averaging or stacking alone were not the best methods of determining probabilities. The calibrated stacked posterior and the calibrated XGBoost anchor were combined with the weight from the validation to get the best calibration. This calibration improvement is not due to test-set tuning, as this weight was measured before the official test partition was examined.

Figure 5 investigates the benefit of further training observations of the probability-level meta-learner and whether its behavior suggests underfitting or overfitting. The number of observations evaluated in the meta-training ranges from about 40,000 to 400,000.

The training curve and the validation curve are close to the top limit over all sample sizes in the accuracy panel. This near overlap suggests that classification was highly accurate and that there does not seem to be a large generalization gap. The vertical axis covers the entire range from zero to one, so minor variations between the curves are compressed in the image. Thus, the resolution itself is not sensitive enough to assess convergence in this high-performance regime.

The log-loss panel gives a more informative evaluation, given that it considers not only if the prediction is correct but also the probability of the correct class. The training log-loss was approximately 0.0110 with 40,000 meta-training observations, while the validation log-loss was approximately 0.0152 with 31,000 validation observations. The initial gap suggests the level of coverage of all class-probability patterns in validation is not good enough in the smaller training set.

The meta-training size was increased to around 100,000 observations; validation log-loss dropped sharply to ∼0.0126, while training log-loss was ∼0.0108. A larger sample size, 200,000 observations, yielded a further small reduction in validation log-loss, further improving the quality and stability of the predicted probabilities.

The two log-loss curves for training and validation data were very close at about 300,000 observations, where they both approached 0.012. The fact that they are so close suggests that the meta-learner was in a stable generalization regime, with no noticeable overfitting. When the number of meta-training observations was increased to 400,000, this seemed to indicate that after about 300,000 observations, the improvement leveled off.

More training logs do not imply more degradation since the log-loss is only slightly increased. Subsets might have more simple or less varied observations that can be fitted more snugly. Larger subsets lead to a wider variety of class-probability combinations, patterns of uncertainty and challenging minority-class examples, and thus to a more realistic training loss. At the same time, the decrease and stabilization of the number of log losses in the validation set show better generalization.

The learning curves do not show the typical signature of overfitting, where the training performance continues to increase and the validation performance continues to decrease. Rather, as more observations are added, the validation log-loss gets lower and closer to the training log-loss. The curves also do not show any signs of severe underfitting since there is good accuracy and log-loss over the range of interest.

Overall, it can be concluded that the leakage-free out-of-fold meta-features contain enough information for the stable fitting of the meta-learner. The number of meta-training observations, at 300,000, seems sufficient for convergence, and the extra observations to 400,000 mostly give stability, not a significant performance benefit. However, if specific types of attacks are rare, it would be better to add more examples of those specific types of attacks than to add more examples of the majority.

#### 4.2.4. Uncertainty and Stability

We provide an evaluation of the sensitivity of XGB-AGMoE to data sampling and model initialization in Table 4. The model showed a high level of accuracy of 0.9926±0.0012 and a high level of weighted-F1 of 0.9919±0.0016 across the 3 seeds. Overall classification performance was also very consistent, with narrow standard deviations, showing the effect of stochastic variations in the experimental pipeline.

Seed 42 was the best-performing seed with the highest score in accuracy (0.993887), macro-F1 (0.851256), and weighted-F1 (0.993681) before evaluation. The lowest corresponding values were produced by Seed 52, with an accuracy of 0.991593, a macro-F1 of 0.802158, and a weighted-F1 of 0.990659. For seed 62, the classification results were intermediate with an accuracy of 0.992347, a macro-F1 of 0.817921, and a weighted-F1 of 0.991435.

Macro-F1 was more variable than accuracy and weighted-F1 but had a higher mean of 0.8238±0.0251. This variation is anticipated for the severe class-imbalanced scenario of the CICIoMT2024, as macro-F1 gives equal importance to each class. A few ARP Spoofing, Penguin Sweep, Recon VulScan, or publish-flood observations can then change macro-F1 significantly but only slightly regarding accuracy. The results for the multiple-seed runs accordingly validate the high stability of the model in terms of the overall test distribution while also exhibiting greater sensitivity of the model towards the recognition of rare classes.

The performance was especially consistent for ranks. Macro-ROC-AUC ranged from 0.999222 to 0.999634, with a mean of 0.9995±0.0002. The very low standard deviation indicates that the model performed consistently in ranking the observations from the correct class higher than the observations from other competing classes. Macro-PR-AUC produced a mean of 0.9197±0.0053, ranging from 0.913777 for seed 52 to 0.924029 for seed 62. In particular, seed 62 had the best macro-PR-AUC, although seed 42 had the best accuracy and macro-F1. This indicates that there was no one seed that was always superior in all performance aspects.

The probability quality was also low throughout the three runs. The mean Brier score was 0.0100±0.0022, while the mean NLL was 0.0199±0.0040. Seed 42 gave the minimum Brier score (0.007531) and minimum NLL (0.015309), which show higher precision of probabilities and higher concentration of confidence of Seed 42. The Briers and NLLs for seeds 52 and 62 were slightly higher but within a small absolute margin.

ECE averaged 0.0034±0.0014. Seed 42 achieved the lowest ECE of 0.001775, followed by seed 62 at 0.003932 and seed 52 at 0.004358. The relative difference in ECE values compared to the accuracy values is larger, but all three values are small, which shows that the overall agreement between predicted confidence and empirical correctness is good.

The mean results should be considered as primary evidence of the robustness of the model, while the seed-42 results are used for the detailed confusion matrix, ROC–PR, and reliability analyses. Seed 42 is not a post hoc best-seed selection; it was the default primary seed, and seeds 52 and 62 were used for stability testing.

Overall, the multi-seed evaluation shows that the XGB-AGMoE exhibits consistently high classification, ranking, and calibration performance. The small differences in Overall Acc, weighted-F1, and macro-ROC-AUC suggest excellent overall reproducibility, with the larger differences in macro-F1 indicating that the performance of rare classes remains sensitive to data composition and to stochastic training effects. The results provide evidence of empirical stability, as only 3 seeds were evaluated, and must not be formally interpreted as a statistical guarantee.

The measurement of the sampling uncertainty of the performance of XGB-AGMoE is shown in Table 5. The corresponding point estimates from the complete official test partition are very close to the corresponding bootstrap means. The bootstrap accuracy of 0.993878 is very close to the corresponding point estimate, 0.993887, and the bootstrap macro-F1 of 0.850143 is close to the corresponding point estimate, 0.851256. The agreement suggests that the results reported are not the result of a small atypical subset of test observations.

The accuracy had a very small 95% confidence interval (0.993446 to 0.994275). It had a total interval width of 0.000829, which indicates that the overall classification rate of the model was estimated with great accuracy. The model achieved consistently high accuracy values even at the lower confidence limit (0.993), indicating the strong overall accuracy.

The bootstrap mean for macro-F1 was 0.850143 with a wider interval of 0.830990 to 0.866878. This larger gap is due to the fact that macro-F1 is more sensitive to rare classes. Changes in a small number of Ping Sweep, Recon VulScan, ARP Spoofing or publish-flood observations can have a significant impact on the bootstrap macro-F1 even though such changes do not have a significant impact on the overall accuracy.

The macro-ROC-AUC estimate was also very stable, with a bootstrap mean value of 0.999629, and its 95% CI was very close to the value of the mean 0.999534–0.999699. This finding gives confidence that this near-perfect ranking performance is not due to a particular test sample. The performance of XGB-AGMoE was always better than the other classifiers in all the bootstrap resamples with respect to the probability given to the correct class when it was one-versus-rest.

Macro-PR-AUC achieved a bootstrap mean of 0.921685, with a 95% confidence interval from 0.904286 to 0.936969. The interval was broader than macro-ROC-AUC, as precision–recall performance is more sensitive to the prevalence of the classes and to false-positive predictions. However, the lower limit of the interval was above 0.90, suggesting excellent precision–recall performance for the 14-class taxonomy.

The uncertainty intervals of the probability-quality measures were also small. The Brier score had a bootstrap mean of 0.007541 and a 95% confidence interval of 0.007099–0.007978. NLL had a mean of 0.015345 and an interval of 0.014400–0.016373. The low values and small ranges suggest that the final probability vectors were accurate and focused on the right classes in repeated test resampling.

ECE produced a bootstrap mean of 0.001788, with a 95% confidence interval from 0.001519 to 0.002044. The full interval remained near zero, which is consistent with the overall results that XGB-AGMoE was well calibrated on average. Furthermore, the bootstrap mean was not far from the full-test ECE of 0.001775, giving further support that the quality of calibration observed was not necessarily due to a subset of the test observations; rather, it was stable.

The interval-width pattern is also informative. Macro-ROC-AUC, Brier score, NLL, and ECE had low sampling variation, while macro-F1 and macro-PR-AUC had higher sampling variation. This difference aligns with the extreme class imbalance: aggregate and probability-weighted measures are heavily affected by the well-represented classes, but macro-averaged measures are still affected by errors in the minority classes.

#### 4.2.5. Computational Performance

The inference benchmark was run on Intel(R) Xeon(R) CPU @ 2.20 GHz, 31.35 GiB system RAM, without GPU acceleration during inference, and Linux-6.12.90+-x86_64-with-glibc2.35 (with Python 3.12.12, scikit-learn 1.6.1, XGBoost 3.1.0, LightGBM 4.6.0, and CatBoost 1.2.8). The processing steps of frozen preprocessing transformations, granular-feature construction, three expert predictions, expert calibration per expert, meta-feature construction, logistic stacking, and final anchored fusion were included in the latency measurements. In total, 30 repetitions were taken per batch size with 5 warm-up runs, and the median elapsed time was reported. Rather, the latency measurements are thus not those of a single expert but are ones for the entire inference pipeline, as specified in the experiment.

For the single-observation and batched inference conditions, the computational efficiency of XGB-AGMoE is compared in Table 6. The model was able to process a single observation with a median time of 21.862 ms, yielding an average sample rate of 45.74 samples per second (sps) with a batch size of 1. This is the lowest response delay at the batch level since a prediction can be returned immediately without awaiting more observations for a batch. Thus, it is the most suitable of the assessed settings for low-volume or latency-oriented online monitoring.

When the batch size is increased to 256, the total batch-processing time is increased to 166.016 ms. The computational cost was spread out over 256 observations, however, resulting in an amortized per-sample latency of 0.6485 ms and a throughput of 1542.02 samples/s. This is about 33.7 times faster than the throughput of single-sample inference and 97.0% faster than per-sample inference latency.

The throughput of the largest batch evaluated, size 1024, was 1896.52 samples/s with a per-sample latency of 0.5273 ms, a 41.5x increase and a 97.6% decrease, respectively, compared to the throughput and latency with a batch size of 1. The results in this section show the significant gains of vectorized and batched prediction in XGB-AGMoE.

The increase in size from 256 to 1024 was smaller than the increase in size from 1 to 256. The throughput rose about 23.0%, and the latency per sample dropped by about 18.7%. This pattern shows that there is not much gain in efficiency with bigger batches. Meanwhile, the total batch delay increased from 166.016 to 539.936 ms, resulting in a balance between the throughput and the time it takes to complete a whole batch.

Thus, the right size of the batch depends on the deployment scenario. Batch size 1 is suitable for making immediate predictions, and it is well suited for low-volume streaming and event-triggered intrusion analysis. The batch size of 256 is a good compromise between throughput and response time. A batch size of 1024 is more suitable for systems that handle high-volume traffic analysis, retrospective processing, or buffered monitoring systems where the maximum throughput is more important than the delay for each individual batch.

Overall, the results demonstrate the potential of individual and high-throughput inference using the frozen XGB-AGMoE model. The previously reported latency, however, depends on the hardware, software, processor load, and implementation used in the experiment. These should thus be viewed as results from the specified experimental environment and not as guarantees for deployment.

### 4.3. Discussion

The model proposed in this study is compared with the previously reported machine learning approaches with CICIoMT2024 in Table 7. XGB-AGMoE achieved an accuracy of 99.39% on the official test, which is competitive in comparison with the existing methods.

Saeed et al. [23] reported the highest accuracy of 99.64% using the Random Forest and other machine learning approaches in a 14-class formulation. When using 19 classes, with the Random Forest method, Dadkhah et al. [22] reported an accuracy of 99.55%. It is proposed that the result of the XGB-AGMoE model is competitive but does not necessarily have the highest accuracy when compared to the results of the models reported by [22,23].

The accuracy of XGB-AGMoE was 2.69 percentage points higher than XGBoost and Random Forest, as reported by Rehman et al. [24] in a 19-class setting. Yacoubi et al. [25] reported an explainable machine learning approach, but no comparable number of classes and accuracy value were provided here, and this is therefore not reported but treated as not reported.

Useful contextual evidence is provided in the table, but the accuracy values are not a controlled head-to-head comparison. Dadkhah et al. [22], Rehman et al. [24], and the present study employed 19-class taxonomies and 14 classes, respectively. The studies can also vary in their division of the train/test set, sampling methods, class distributions, preprocessing, leakage controls, and hyperparameter-selection methods. The significance of any differences of less than a few tenths of a percentage point ought not to be attributed to a definite superiority without repeating all methods under a similar protocol.

The contribution of XGB-AGMoE is not only on the total accuracy metric. It is a proposal of a combination of continuous representation, heterogeneous boosting experts, held-out probability calibration per-emp, leakage-free out-of-fold stacking, and validation-selected XGBoost anchoring. It was tested on a disjoint official test partition, and the study reported class-balanced discrimination and calibration metrics as well as accuracy. These included a macro-F1 of 85.13%, a macro-ROC-AUC of 99.96%, a macro-PR-AUC of 92.14%, a Brier score of 0.007531, an NLL of 0.015309, and an ECE of 0.001775.

This more comprehensive assessment is crucial because this can lead to a misleadingly accurate aggregate score, while the classes on a highly unbalanced intrusion-detection dataset may be trivially easy to classify correctly. So the major benefit of XGB-AGMoE is not that it has the highest reported accuracy, but that it classifies the classification results with leakage-controlled evaluation and performs an explicit analysis of the minority class, probability calibration, uncertainty assessment, and the stability evaluation of multiple seeds.

## 5. Ablation and Baseline Analysis

The contribution of the principal components of XGB-AGMoE and the comparison of all the models with individual experts and alternative probability-combination strategies is presented in Table 8. XGB-AGMoE achieved the highest accuracy of 0.993887, macro-F1 of 0.851256, and macro-ROC-AUC of 0.999634. It also obtained the lowest Brier score of 0.007531, NLL of 0.015309, and ECE of 0.001775. The results suggest that the proposed model has the best overall performance in terms of classification accuracy, class-wise discrimination, and probability reliability.

### 5.1. Effects of Calibration and Adaptive Anchoring

Stacking without calibration had an accuracy of 0.990800 and a macro-F1 of 0.758417. Held-out probability calibration and validation adaptive XGBoost anchoring improved accuracy by 0.003087 (0.31% points) and macro-F1 by 0.092839 (9.28% points). Calibration and anchoring primarily helped class-balanced performance, evidenced by the greater improvement in macro-F1.

Additionally, XGB-AGMoE outperformed uncalibrated stacking in terms of macro-ROC-AUC (0.999634 vs. 0.994604) and macro-PR-AUC (0.921352 vs. 0.880965). Furthermore, it improved the Brier score by about 48.6%, the NLL score by about 59.4%, and the ECE score by about 65.8%. The results show that it is important to build the final model using the calibrated probabilities for discrimination and for confidence reliability.

### 5.2. Comparison with Probability Averaging

The raw expert average has an accuracy of 0.991907 and a macro-F1 of 0.815854, while the calibrated expert average has an accuracy of 0.991800 and a macro-F1 of 0.813017. XGB-AGMoE outperforms the calibrated averaging method by 0.038239 and the raw averaging method by 0.035402 in terms of macro-F1. The finding suggests that a learned class-dependent combination of expert outputs, followed by adaptive XGBoost anchoring, is better than giving all experts equal weights.

The simple average with some calibration improved the Brier score and the NLL, with scores changing from 0.011183 and 0.021359 to 0.010980 and 0.019943, respectively. However, ECE increased slightly from 0.004437 to 0.004627. The result shows that the calibration of each expert does not maximize the average of each calibration metric after averaging their probabilities.

The Brier score was lowered by about 31.4%, the NLL score by 23.2%, and the ECE score by 61.6% compared to the calibrated average of the experts. So, its usefulness is not due to calibration alone but also due to the stacking relationships learned and the anchor validated to be adaptive to the validation sample.

The calibrated expert average had the highest macro-PR-AUC of 0.926032; the raw expert average was 0.924321, followed by XGB-AGMoE (0.921352). So, the proposed model was not the best on all metrics. It has a lower macro-PR-AUC but offers significant improvements in Brier, NLL, and ECE, and higher values of accuracy, macro-F1, and macro-ROC-AUC. This is also consistent with choosing the anchor weight based on the validation macro-F1 rather than optimizing the actual macro-PR-AUC.

### 5.3. Comparison with Individual Boosting Experts

The calibrated CatBoost had an accuracy of 0.990787 and a macro-F1 of 0.790624, while the calibrated LightGBM had an accuracy of 0.991400 and a macro-F1 of 0.789944. When compared to CatBoost and LightGBM, XGB-AGMoE achieved significant gains of 0.060632 and 0.061312 in macro-F1, respectively. Moreover, it achieved higher macro-ROC-AUC and macro-PR-AUC values and much lower Brier score, NLL score, and ECE score than either of the two experts.

The improvements facilitate the use of heterogeneous boosting learners. CatBoost is provided with the mixed continuous–granular representation, while LightGBM is provided with a different tree-growth strategy with a standardized continuous representation. While none of the experts duplicate the entire model, their calibrated probabilities add value to the meta-learner.

### 5.4. Comparison with the Logistic Feature Baseline

The logistic feature baseline had the lowest performance with an accuracy of 0.981620, a macro-F1 of 0.626742, and a macro-PR-AUC of 0.653136. XGB-AGMoE achieved a 1.23% accuracy gain and a 0.224514 macro-F1-score gain, which is equivalent to a 22.45% score gain. It also reduced the Brier score from 0.031451 to 0.007531 and NLL from 0.100546 to 0.015309.

The results can be seen in the weaker logistic baseline, which is a clear indication that the decision boundaries of CICIoMT2024 cannot be well captured by a simple linear mapping of the original features. The modeling of the complex relations between the traffic variables requires nonlinear tree interaction, heterogeneous experts, and fusion at the probability level.

### 5.5. Contribution of Granular Features

Removing granular features reduced accuracy from 0.993887 to 0.991833 and macro-F1 from 0.851256 to 0.781029. The macro-F1 decrease of 0.070227 (7.02 percentage points) suggests that the granular representation helps class-balanced recognition to a greater extent. The NoGranular GMoE also achieved a lower macro-ROC-AUC (0.999232) and macro-PR-AUC (0.879298). In addition, removing the granular representation increased the Brier score from 0.007531 to 0.012696, NLL from 0.015309 to 0.031424, and ECE from 0.001775 to 0.003184. The complete model was able to improve Brier by approximately 40.7%, NLL by approximately 51.3%, and ECE by approximately 44.3% relative to this ablation. The results indicate the usefulness of supplementing the standard descriptors of continuous variables with quantile-bin identity, granular confidence, and empirical bin-frequency descriptors. The granular features offer distribution-aware information, which is not explicitly captured by the original continuous information, hence giving better discrimination for minority classes and probability quality.

The same protocol baseline and ablation results are summarized in Figure 6. The macro-F1-score of XGB-AGMoE (0.8513) was higher than the raw expert averaging, the calibrated expert averaging, NoGranular GMoE, uncalibrated stacking, CatBoost, LightGBM, and the logistic feature baseline. The granular features were removed to get macro-F1 = 0.7810, and the raw probabilities were stacked to get macro-F1 = 0.7584. These changes reflect the contributions of the granular representation and held-out calibration. Calibrated XGBoost, however, had a higher canonical macro-F1 of 0.8764 and continues to be a top individual component baseline. The probability measures show a balance, rather than one measure consistently dominating the others. While not as good as the calibrated and raw experts’ average, XGB-AGMoE slightly outperformed the displayed ensemble alternatives in terms of calibration. Its ECE was 0.001775, and its Brier score was 0.007531. The results consequently validate the idea of anchoring with validation control and evaluation with probabilities and demonstrate that the contribution of stacking is dependent on the chosen validation split and evaluation goal.

## 6. Conclusions

This paper built an XGB-AGMoE framework for 14-class intrusion detection in IoMT WiFi–MQTT traffic, which was based on XGBoost and validated adaptively by the test set. The validation partition chose the final posterior with 20% calibrated stacked probability and 80% calibrated XGBoost probability, as per the predefined seed-42 protocol. On the official test sample, this configuration achieved an accuracy of 0.993887, weighted-F1 of 0.993681, macro-F1 of 0.851256, macro-ROC-AUC of 0.999634, macro-PR-AUC of 0.921352, Brier score of 0.007531, NLL of 0.015309, and ECE of 0.001775. The ablation analysis demonstrates the value of granular descriptors, held-out probability calibration, and leakage-free stacking as compared to their reduced counterparts. The results of the classes, however, indicate that there was no uniformity in performance. Some attacks, such as DDoS Publish Flood, Recon VulScan, Ping Sweep, and ARP Spoofing, were still more difficult to detect than the more common classes of denial-of-service attacks. Furthermore, calibrated XGBoost performed better than the fused posterior on some seed-42 measures, and equal expert averaging resulted in a slightly higher macro-PR-AUC. XGB-AGMoE is, therefore, a validation-controlled model that allows the best expert to be chosen only when backed by development data, and not one that will always rank best on all splits. The study considers only CICIoMT2024, computationally bounded partitions, and small test support for a few categories of attacks. Sample-level bootstrap intervals and the three-seed experiments provide estimates of the variation within the dataset; they are not assessments of generalization to previously unseen institutions, devices, time periods, or traffic distributions. The framework needs to be tested on independent IoMT datasets, explore anchor selection validation at different time levels and device levels, and test latency and memory usage on target medical edge devices.

## Figures and Tables

**Figure 1:**
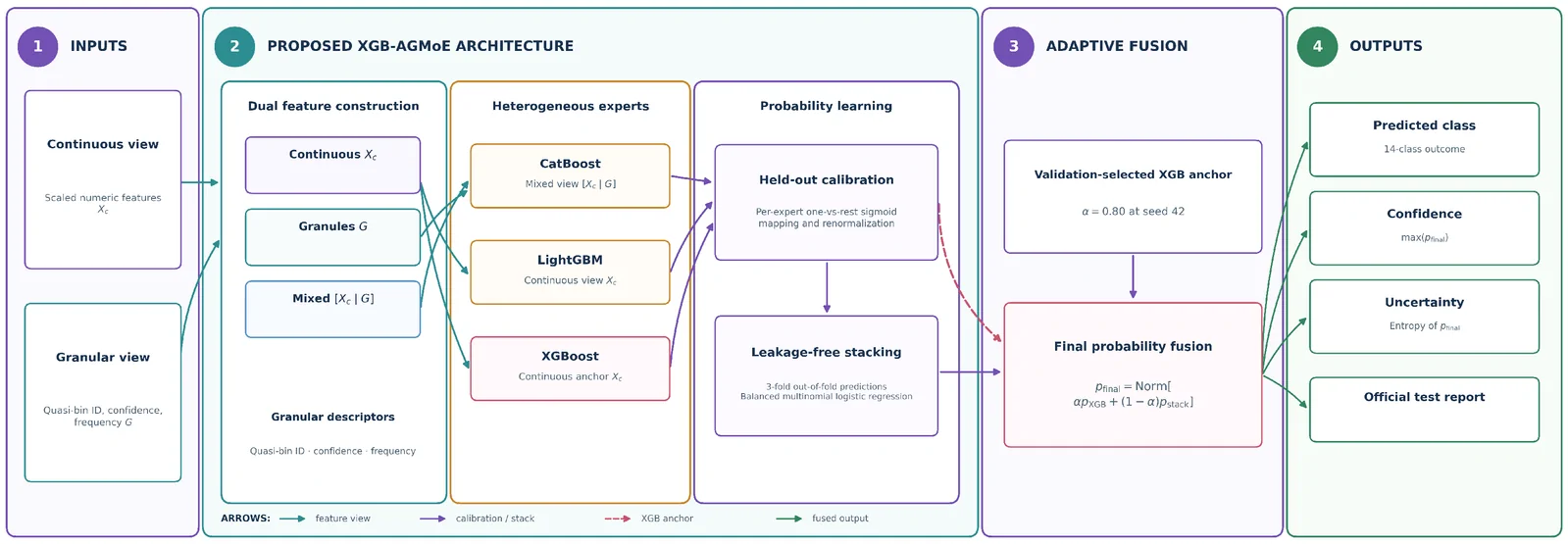
Architecture of the proposed XGB-AGMoE framework.

**Figure 2:**
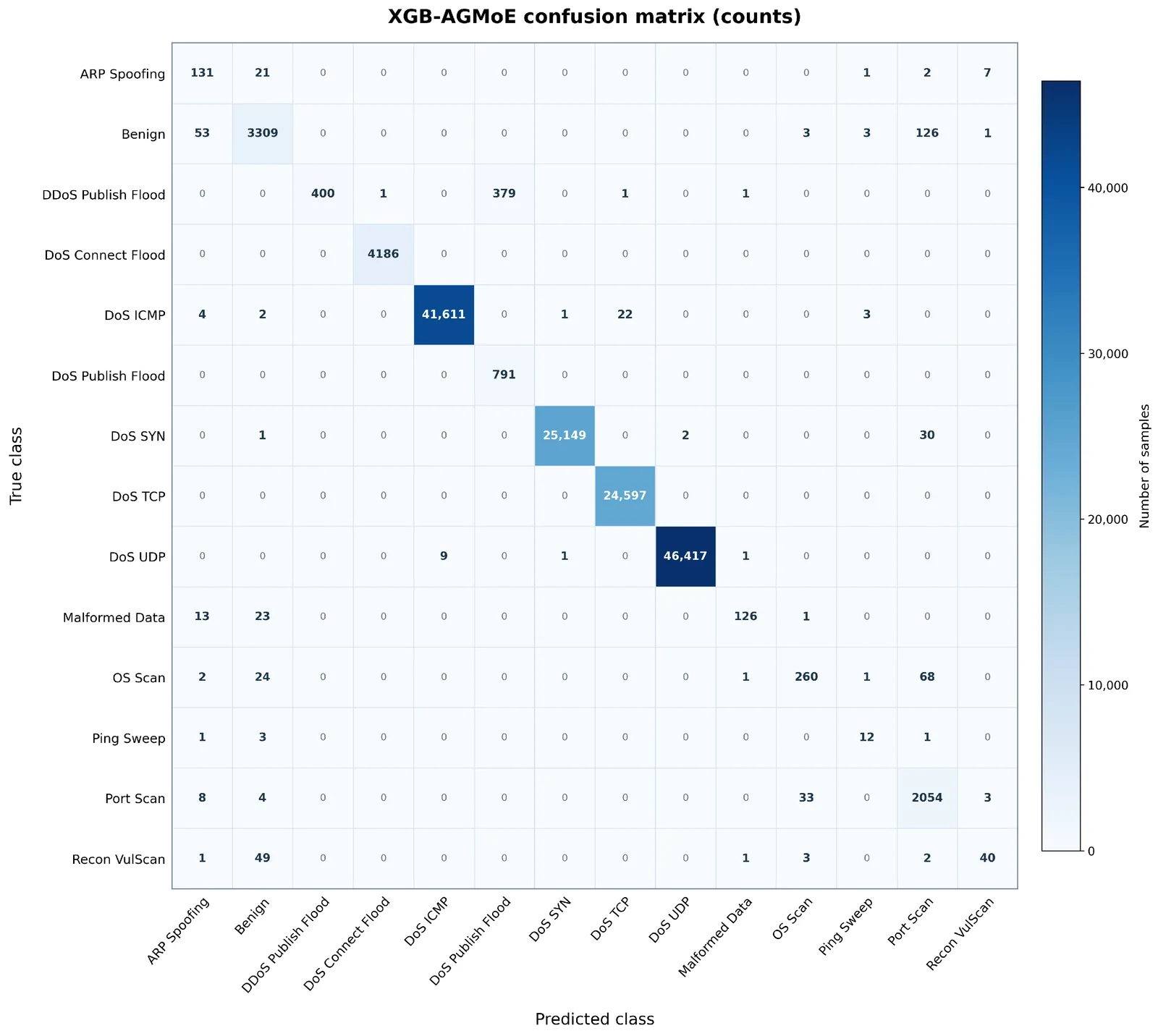
XGB-AGMoE count and row-normalized confusion matrices.

**Figure 3:**
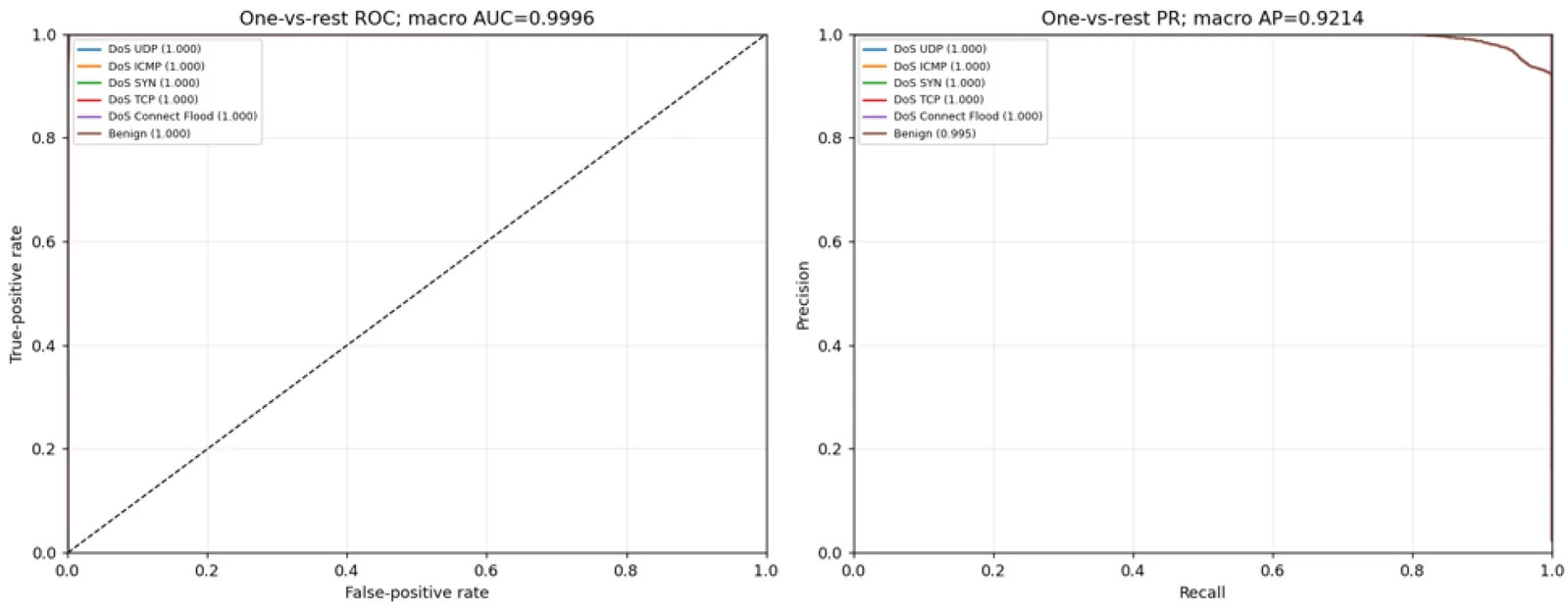
One-versus-rest receiver operating characteristic and precision–recall curves of the proposed XGB-AGMoE model on the official CICIoMT2024 test partition.

**Figure 4:**
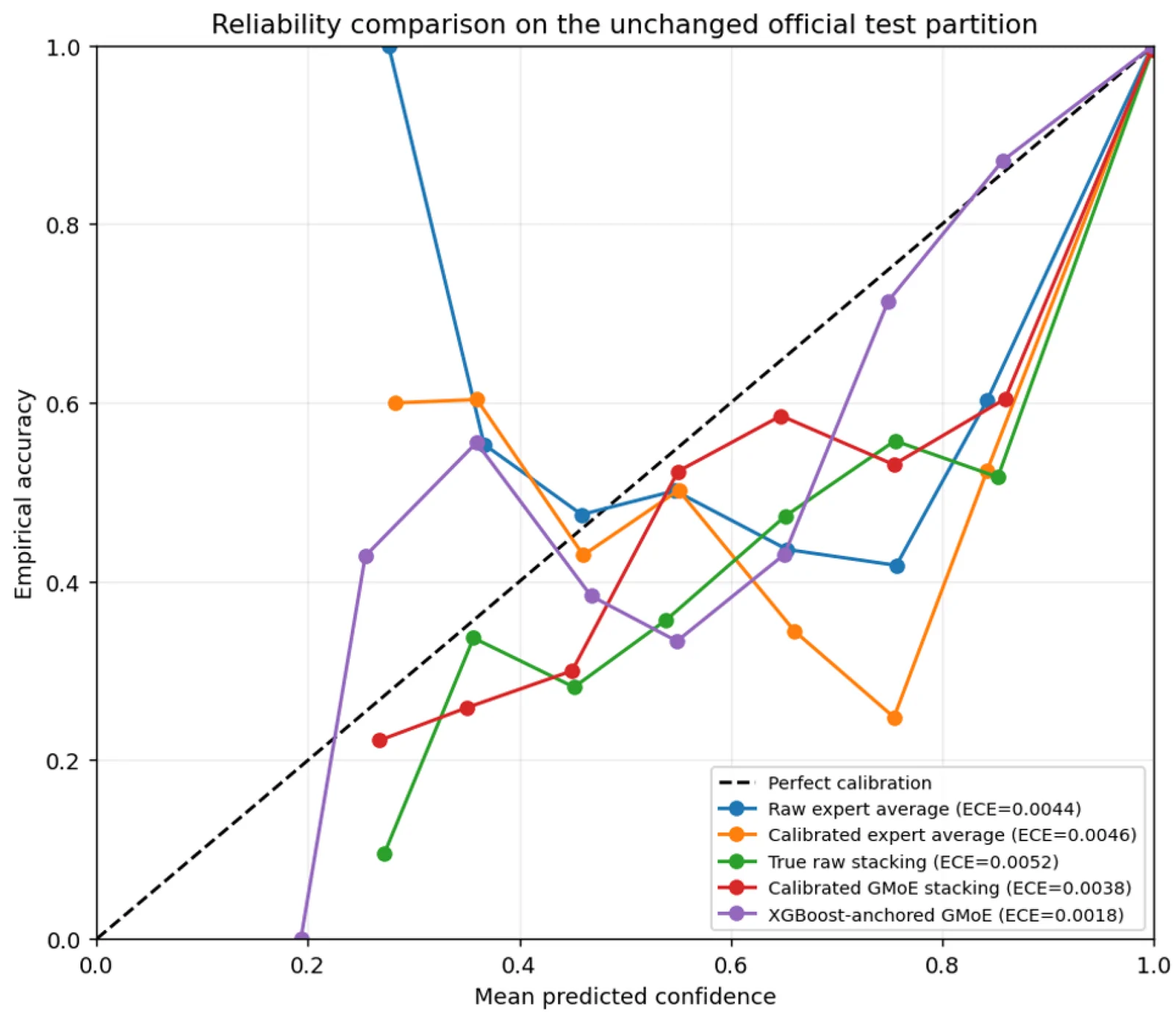
Reliability diagrams comparing probability-fusion strategies on the unchanged official CICIoMT2024 test partition.

**Figure 5:**
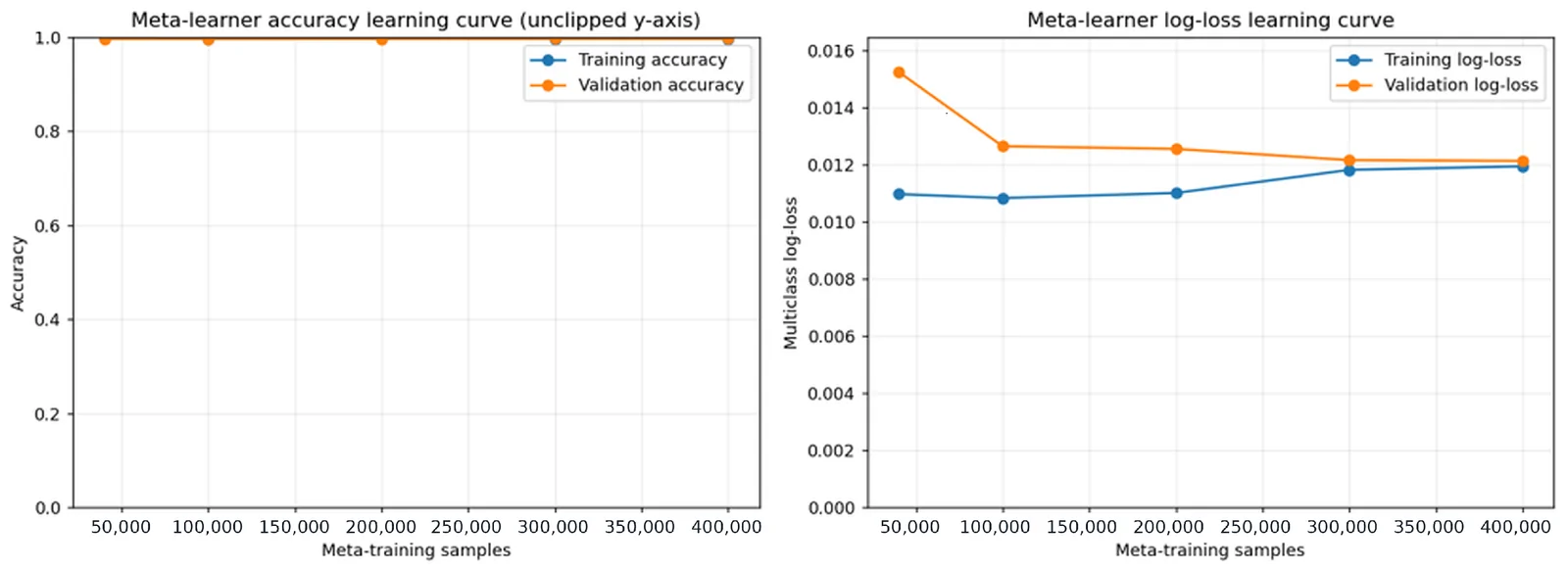
Learning curves of the calibrated XGB-AGMoE meta-learner across increasing meta-training sample sizes.

**Figure 6:**
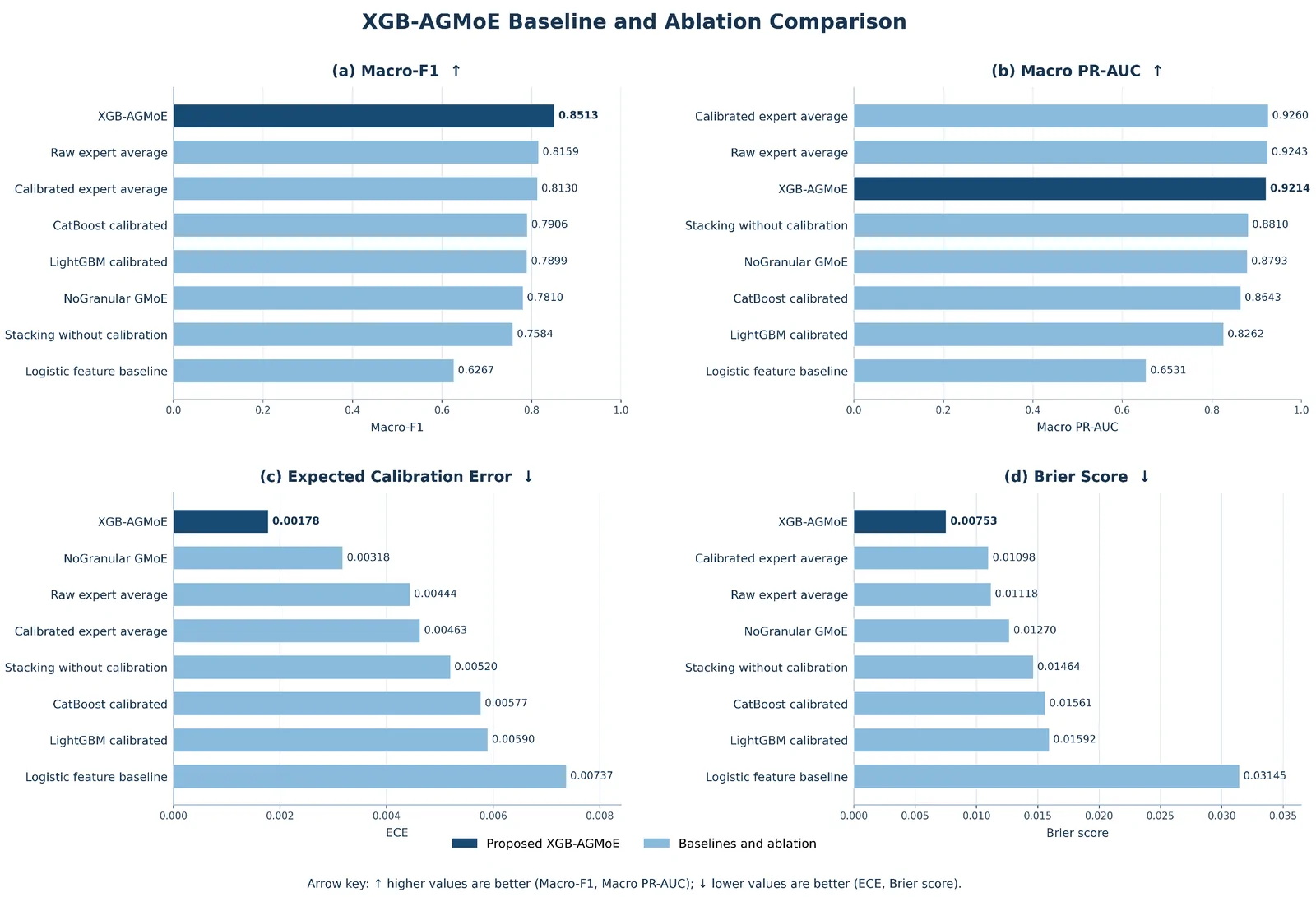
Baseline and ablation comparison of the proposed XGB-AGMoE model on the official CICIoMT2024 test partition.

**Table 1:** Leakage-controlled experimental partitioning protocol and the role of each data subset in XGB-AGMoE development and evaluation.

Partition	Rows Used	Definition	Purpose
Base training	500,000	76.5% of original training before cap	Expert fitting
Calibration pool	80,000	8.5% of original training before cap	50,000-row calibrator fitting cap
Validation	100,000	15% of original training before cap	Anchor selection and validation analyses
Official test sample	150,000	Separate supplied test parquet	Final evaluation only

**Table 2:** Classification, ranking, and probability-calibration performance of the proposed XGB-AGMoE model on the official test set.

Metric	XGB-AGMoE
Accuracy	0.993887
Macro-F1	0.851256
Weighted-F1	0.993681
Macro-ROC-AUC	0.999634
Macro-PR-AUC	0.921352
Brier	0.007531
NLL	0.015309
ECE	0.001775
MCE	0.220490

**Table 3:** Class-wise classification performance of the proposed XGB-AGMoE model on the official CICIoMT2024 test partition.

Class	Precision	Recall	F1-Score	Support
ARP Spoofing	0.61502	0.80864	0.69867	162
Benign	0.96304	0.94678	0.95484	3495
DDoS Publish Flood	1.00000	0.51151	0.67682	782
DoS Connect Flood	0.99976	1.00000	0.99988	4186
DoS ICMP	0.99978	0.99923	0.99951	41,643
DoS Publish Flood	0.67607	1.00000	0.80673	791
DoS SYN	0.99992	0.99869	0.99930	25,182
DoS TCP	0.99907	1.00000	0.99953	24,597
DoS UDP	0.99996	0.99976	0.99986	46,428
Malformed Data	0.96923	0.77301	0.86007	163
OS Scan	0.86667	0.73034	0.79268	356
Ping Sweep	0.60000	0.70588	0.64865	17
Port Scan	0.89969	0.97716	0.93683	2102
Recon VulScan	0.78431	0.41667	0.54422	96
Macro average	0.88375	0.84769	0.85126	150,000
Weighted average	0.99483	0.99389	0.99368	150,000

**Table 4:** Multi-seed stability of the proposed XGB-AGMoE model on the official CICIoMT2024 test partition.

Seed	Accuracy	Macro-F1	Weighted-F1	Macro-ROC-AUC	Macro-PR-AUC	Brier	NLL	ECE
42	0.993887	0.851256	0.993681	0.999634	0.921352	0.007531	0.015309	0.001775
52	0.991593	0.802158	0.990659	0.999222	0.913777	0.011182	0.022691	0.004358
62	0.992347	0.817921	0.991435	0.999518	0.924029	0.011364	0.021803	0.003932
Mean ± SD	0.9926±0.0012	0.8238±0.0251	0.9919±0.0016	0.9995±0.0002	0.9197±0.0053	0.0100±0.0022	0.0199±0.0040	0.0034±0.0014

**Table 5:** Bootstrap estimates and 95% confidence intervals for the performance of XGB-AGMoE on the official CICIoMT2024 test partition.

Metric	Bootstrap Mean	95% CI Lower	95% CI Upper
Accuracy	0.993878	0.993446	0.994275
Macro-F1	0.850143	0.830990	0.866878
Macro-ROC-AUC	0.999629	0.999534	0.999699
Macro-PR-AUC	0.921685	0.904286	0.936969
Brier	0.007541	0.007099	0.007978
NLL	0.015345	0.014400	0.016373
ECE	0.001788	0.001519	0.002044

**Table 6:** Inference latency and throughput of the frozen XGB-AGMoE model at different batch sizes.

Batch Size	Median Batch Latency (ms)	Median Sample Latency (ms)	Throughput (Samples/s)
1	21.862	21.862	45.74
256	166.016	0.6485	1542.02
1024	539.936	0.5273	1896.52

**Table 7:** Comparison of XGB-AGMoE with previously reported machine learning approaches evaluated using the CICIoMT2024 dataset.

Reference	Method	Number of Classes	Reported Accuracy
Dadkhah et al. (2024) [22]	Random Forest	19	99.55%
Saeed et al. (2025) [23]	Random Forest and other machine learning methods	14	99.64%
Rehman et al. (2025) [24]	XGBoost and Random Forest	19	96.70%
Yacoubi et al. (2026) [25]	Explainable machine learning	NR	NR
**This study**	**XGB-AGMoE: Granular features, CatBoost/LightGBM/XGBoost experts, held-out calibration, leakage-free OOF logistic stacking, and validation-adaptive XGBoost anchoring**	**14**	**99.39%**

NR: Not reported in the cited study.

**Table 8:** Comparative and ablation analysis of the proposed XGB-AGMoE model on the official CICIoMT2024 test partition.

Model	Accuracy	Macro-F1	Macro-ROC-AUC	Macro-PR-AUC	Brier	NLL	ECE
XGB-AGMoE	0.993887	0.851256	0.999634	0.921352	0.007531	0.015309	0.001775
Stacking without calibration	0.990800	0.758417	0.994604	0.880965	0.014639	0.037706	0.005196
Calibrated expert average	0.991800	0.813017	0.999553	0.926032	0.010980	0.019943	0.004627
Raw expert average	0.991907	0.815854	0.999522	0.924321	0.011183	0.021359	0.004437
CatBoost calibrated	0.990787	0.790624	0.997941	0.864329	0.015605	0.052357	0.005766
LightGBM calibrated	0.991400	0.789944	0.996438	0.826182	0.015917	0.065667	0.005900
Logistic feature baseline	0.981620	0.626742	0.992033	0.653136	0.031451	0.100546	0.007370
NoGranular GMoE	0.991833	0.781029	0.999232	0.879298	0.012696	0.031424	0.003184

## Data Availability

Dataset available at https://www.unb.ca/cic/datasets/iomt-dataset-2024.html; accessed on 1 March 2025.
